# Improving the Effectiveness of Healthcare: Diagnosis-Centered Care Vs. Person-Centered Health Promotion, a Long Forgotten New Model

**DOI:** 10.3389/fpubh.2022.819096

**Published:** 2022-05-16

**Authors:** Andrey Martyushev-Poklad, Dmitry Yankevich, Marina Petrova

**Affiliations:** Federal Research and Clinical Center of Intensive Care Medicine and Rehabilitology, Moscow, Russia

**Keywords:** healthcare organizational model, clinical effectiveness, cost effectiveness, biomedical paradigm, biopsychosocial paradigm, model of care, person-centered model of care

## Abstract

Performance of healthcare can be measured as its ability to restore and preserve health with acceptable costs for the society. Under the current prevalence of chronic disease, medical care (the major content of healthcare) underperforms in all key indicators: clinical effectiveness, benefit/risk ratio of interventions, cost/benefit ratio, and general population health. In Russia key performance indicators (KPI) of healthcare do not allow effective decision-making; a similar situation is seen worldwide: most KPIs are either focused on the process (not results) of medical care, or depend on efforts out of control of healthcare decision-makers.

The key root factors limiting clinical effectiveness and cost-effectiveness of healthcare are reactive diagnosis-centered organizational model of care and the underlying biomedical paradigm, generally inadequate in chronic diseases. They make healthcare intervene too late, use less effective prevention and treatment instruments, and be in a state of resource scarcity. In Russia there is also a lack of interdisciplinary and interagency cooperation essential for health preservation and promotion.

Performance of healthcare system in overcoming the chronic disease epidemic can be improved through supplementing the current ‘reactive’ organizational model with preventive person-centered model based on the biopsychosocial paradigm. Enabling patients for early lifestyle-based interventions, the core P4 medicine approach, should prevail in managing chronic disease. Communication and information technologies should allow fast scaling up of the best person-centered practices.

## Introduction

By definition, a health system is the aggregate of all public and private organizations, institutions, and resources mandated to improve, maintain or restore health (1). In the former USSR, healthcare system achieved the level of universal health coverage and was a benchmark for many other countries. After dissolution of the USSR the system rapidly degraded in Russia and all other post-Soviet states (2).

Healthcare is both one of the core branches of economy and a major (together with education) determinant of human capital. However, many lay people and even decision makers in Russia tend to reduce healthcare to medical care. What's more, it is not generally understood that problems of health (“a state of complete physical, mental and social wellbeing”, the WHO definition) extend far beyond healthcare, which is mostly responsible for medical care in diseases.

The issues of health preservation and promotion, of managing individual and public health, of increasing lifespan and healthspan, of improving health quality are inherently interdisciplinary. They relate to a wide range of sciences and practices: demography, sociology, psychology, education and pedagogics, public governance, fitness and sports, tourism and recreation, culture and arts, agriculture and food industry, ecology and environmental protection, labor and employment, etc. However, in Russia most concepts, studies and projects related to health lack interagency coordination and cooperation; decision makers and experts usually fail to raise system-based, bird view questions, let alone practicing system approach to health issues. Here we attempt to discuss the issues of health care, preservation and promotion as interdisciplinary, as those requiring united efforts of experts from various fields.

From regulatory standpoint, when evaluating performance of health care we have to consider the formal criteria, KPI established for health system in general and medical care in particular. And with that, performance of the whole heath system is more important than that of separate medical interventions or technologies, since there are so many complex and diverse determinants of personal health over the lifespan. For example, high-technology care (e.g., an expensive surgery) may become unnecessary in case of effective prevention, and useless if the patient faces poor social and living conditions after discharge or has no access to rehabilitation.

What health system performance criteria are used in Russia? How helpful are they in decision making at different levels: national, regional, organizational and personal? The answer is amazing.

Strategic documents that establish national policies in health care [State Program of the Russian Federation “Development of Healthcare”, (3, 4)], reflect its interagency status and define some key social indicators of performance: mortality (general and from different causes), and life expectancy. Certainly, these KPI cannot depend solely on the Russian Health Ministry. However, the Program states nothing about the contribution and responsibilities of other parties: the Ministries of Foreign Affairs, of Defense, of Agriculture, of Labor, of Education and Science, etc.

It is also quite unexpected to find that performance indicators established in Russia for the health system and medical organizations at all levels are either absent or fail to adequately evaluate the quality of services – that is, do not allow effective decision making (5, 6). This means that at the national level the Russian Health Ministry is responsible for only the *process* of providing medical care, but not for *the results* of medical care.

Indicators that are used at the level of medical organizations (MO) to assess the quality of medical care in fact characterize (1) openness and accessibility of information about the MO; (2) accessibility of medical services and the level of convenience; (3) waiting time in the queue before medical service is provided; (4) benevolence, politeness and competence of the staff; (5) satisfaction with the quality of service (Russian Health Ministry Order No.810a of 31October, 2013). In other words, performance indicators of medical organizations reflect neither the state of patients' health nor even the results of medical services, but the process of providing the services. That is, KPI in healthcare are close to those in service sector.

The lack of assessment criteria and performance indicators in the health system and medical care makes it very difficult to develop an adequate strategy of their development.

In this hypothesis and theory article we are discussing the issues of health care, health saving and health promotion as interdisciplinary, by answering the following three questions:

1. What Facts Point at low Performance of Today's Healthcare Systems?
2. How Is Performance of Healthcare Systems Related to the Prevailing Scientific Paradigm and Organizational Model of Health Care?
3. How can Performance of Healthcare System be Improved Through Shift or Evolution of the Paradigm and Organizational Model?

In answering these questions we are describing a new model of health promotion which is actually a forgotten model that used to exist long before the 20^th^ century. Today it can be re-introduced at a new level with the help of digital technologies.

## Question 1. What Facts Point at Low Performance of Today's Healthcare Systems?

In discussion of healthcare performance we consider not only Russia but also high income economies.

There are three terms used to characterize performance: effectiveness, efficacy, and efficiency (7). Effectiveness: the extent to which a specific intervention, procedure, regimen or service, when deployed in the field in routine circumstances, does what it is intended to do for a specified population. Efficacy: the extent to which a specific intervention, procedure, regimen or service, produces the intended result under ideal conditions. Efficiency: the capacity to produce the maximum output for a given input.

Measures of performance are determined by the system's objectives, which are, in turn, set by the subject of management, or decision maker. There are several groups of stakeholders, potential subjects of management and decision makers in the health system: (1) ordinary citizens (end users), (2) health professionals and service providers, (3) governmental and public bodies of different level (national, regional), (4) manufacturers and distributors of medicines and health-related supplies, equipment, etc.

Who are the major decision makers and the subjects of management in healthcare system? In answering this question we have to consider that health-related businesses play a significant part in today's economy. Notably, more than 22.5% of the world health expenditures ($1,2 trillion) are accumulated by pharmaceutical companies which can influence the expert community and even national and global policies.

As we have demonstrated, in Russia there is actually no explicit goal-setting that would adequately reflect performance of medical organizations and health authorities (5, 6). This means that goal-setting for decisions, at least in Russia, is performed “by default”, based on the organizational model and the paradigm embedded in the healthcare system. This is the worldview, the system of concepts that determines the framework for goal-setting, the limits of possibilities, cause-effect relations; together they allocate participants' roles, set the principles, rules and algorithms of their functioning. Other important aspects in goal-setting are motivation of decision makers (for example, financial and commercial interests), available technologies and resources (for example, possibility of automation), socioeconomic and organizational context (for example, the management structure in a particular industry).

We will come back to the evidence of low performance after a brief look at healthcare performance indicators used outside of Russia. These KPI were reviewed in 2018 by Perić et al. (8), divided in clusters (9) and used to cluster health systems of OECD countries (10). A total of existing 361 KPI of health systems can be divided in several domains; most important domains and indicators within them are: access to care (like insurance coverage), efficiency (like health care expenditure, number of hospital beds), quality of care (like rates of hospital-acquired infections, infant and maternal mortality), equity, health status (like life expectancy, healthy life years), and health determinants (like body mass index, smoking and unemployment rates).

If we look at the health system in Russia, it actually uses most of the KPI adopted worldwide, but still the local experts consider them marginally useful for decision-making, especially at micro- (personal) and strategic levels. Here is why, in our opinion:

(1) At micro-level, in decision making doctors are bound by disease-specific guidelines, while patients are influenced a lot by their health-related education and motivation to change everyday behavior toward healthy lifestyle. None of the common KPI is relevant to make right decisions, except (to very little extent) body mass index and smoking.
(2) At macro-(strategic) level, the listed KPIs are either too late to make timely regulatory decisions (life expectancy, Healthy Life Years) or require resources and coordinated efforts of multiple governmental bodies (insurance, poverty, unemployment).

That is, problems with adequate goal-setting within health system can be expected not only in Russia but in most countries. We consider that this is closely related to the scientific paradigm and health system organizational model. These two topics are very rarely discussed by both policymakers and academic professors. And yet it is the scientific paradigm and healthcare organizational model that determine goal-setting at all levels of the system (from general practitioner to regulatory authorities), as well as the range of the expected and attained results.

A scientific paradigm that underlies a healthcare system has to address certain key questions: “what is the human being?”, “what is health?”, “what are health determinants in general and in case of a particular person?”, “what are the principles and exact algorithms/ instruments of health restoration, preservation and promotion?” These interdisciplinary questions relate to physiology and philosophy, psychology and sociology, and many other fields; but they all should lead to and suggest very concrete practical solutions.

For example, since human nature is a combination of physical, mental and social (spiritual) aspects, human health also has these aspects; these aspects of health need a measure so that patients are properly educated, routed, and receive relevant help; health determinants should influence the range of instruments used for diagnosis, treatment and prevention, and so forth.

Today's medicine is mostly a product of the “biomedical paradigm” formulated in the late 19^th^ century and influenced by the microbial theory of infectious diseases. Its advantages and limitations had not been actively discussed until about 20 years ago (11, 12). “This model conceptualized disease as deviation from normal biological functioning owing to biological determinants, described in the language of the basic biomedical sciences, including anatomy, physiology and molecular biology… The biomedical model directed the physician to correct disease and restore normal functioning.” (12).

The central concepts of medical practice such as “nosology” and “medical diagnosis” naturally derive from the biomedical paradigm. Making the right diagnosis is the doctor's key professional task; and therefore the prevalent organizational model of health care can be defined as “diagnosis-centered”.

In real everyday practice it is absence of a medical diagnosis that distinguishes a “practically healthy” from an unhealthy person. And doctor's duty is to prescribe treatment in line with clinical guidelines specific for the patient's diagnosis. “Diagnosis-centeredness” permeates both medical practice and regulatory policies in healthcare. It determines the intrinsic logic, principles and exact algorithms of all interactions between the patient and health system – and correspondingly, it sets the stage for the system's limitations and problems.

What are the consequences of biomedical paradigm and diagnosis-centered organizational model being prevalent in healthcare system? In acute diseases and conditions this model allows their timely identification and effective treatment. However, it turns out to be failing in the face of the chronic disease epidemic. This holds true for both Russia and all the world's developed economies. What is this verdict based upon?

### Evidence 1. Analytical Opinion About Performance of Healthcare Systems in the EU and USA

In 2011 EMEA experts named the current situation as a ‘crisis’ and listed the following drivers of healthcare costs, which can also be seen as the causes of low performance (low efficiency) (13):

- Aging populations and the related rise in chronic disease;
- The spread of unhealthy lifestyles;
- Overly bureaucratic systems;
- Increased specialization in medicine;
- Growing demand by an educated public for access to expensive modern medicine;
- Legacy priorities and financing structures that are ill-suited to today's requirements (i.e. to prevalence of chronic diseases).

According to one of the experts, “Healthcare systems in Europe look like they are designed for the 1950s. They are oriented around acute care. Medical education is oriented around hospitals. Payment systems are oriented around particular interventions”. As a result, the demands on the healthcare system are starting to exceed its capacity to provide appropriate treatment and care.

According to reviews from the USA (14, 15), in 2008 “wasteful spending in the US health system has been calculated at up to $1.2 trillion of the $2.2 trillion spent nationally, more than half of all health spending.” The top three areas of wasted spending are defensive medicine ($210 billion annually), inefficient claims processing (up to $210 billion annually), and care spent on preventable conditions related to obesity and overweight ($200 billion annually).

Thus, even in the leading world economies experts provide evidence of their national healthcare systems being ineffective and inefficient. The structure of healthcare systems very poorly suits today's challenges and problems of public health (the challenge of chronic disease) and cannot cope with them, in part due to lack of efficiency.

Analysts suggest the following causes of healthcare inefficiency in the USA (compared to other developed economies) (16):

1. Higher prices of pharmaceuticals and medical services.
2. Less efficiency in utilization of facilities and equipment.
3. Higher cost of insurance administration.
4. Substitution of higher-cost services for lower-cost options with little additional benefit.
5. High prevalence of obesity (due to lack of prevention and lifestyle-focused interventions).
6. Low productivity gains due to payment policies that reward providers based on volume of services rather than the value of care.

### Evidence 2. “Healthcare Costs / Lifespan” Ratio

One of generally accepted indices of healthcare performance is relation of per capita health expenditure and life expectancy. Two countries close in life expectancy, the USA and Cuba, feature 20-fold difference in per capita healthcare expenditure (17). This fact suggests that there is a huge potential for improving cost effectiveness of healthcare, and it can be used with a different organizational model.

### Evidence 3. Iatrogenesis as the Cause of Deaths in the USA and EU

Another important indicator of healthcare performance is deaths related to incorrect or inadequate medical interventions. Unfortunately, this data is not available for Russia. Detailed studies of this problem in the USA were published in 2001 and 2015 (18, 19): they suggest that iatrogenic causes account for over 250 thousand deaths annually, which makes then the third top cause of mortality. Many of them are due to adverse drug reactions (ADR), considered preventable in 70% cases (20). Annual mortality from ADR in the EU amounts to 197 thousand cases; this is the firths top cause of hospital deaths. ADRs are responsible for 5% of all hospital admissions; the total annual cost to society of ADRs in the EU is €79 billion (21).

Thereby, mortality from incorrect medical interventions or ineffective standards of prevention and treatment make a significant contribution to overall mortality, which is documented for top developed economies. This let us conclude that a major part of large healthcare budgets is spent unproductively or even counterproductively.

### Evidence 4. Effectiveness of Pharmaceuticals

Medicinal products are the most widely used and important tools in medicine and healthcare in general. How effective are they?

Active introduction of evidence based medicine (EBM) in healthcare systems started in the early 1990s. Today EBM principles can be reduced to the following: “any medical intervention, including a drug treatment, should be used in accordance with its efficacy data obtained through controlled clinical trials”. EBM is the cornerstone of international and national clinical guidelines on diagnosis and treatment of diseases, and Russia is no exception.

Unfortunately, clinical practice often challenges both advantages of EBM and practical utility (i.e. clinical effectiveness) of approved pharmaceuticals in real life. Unsurprisingly, in 2015 even in the US hospitals in about 70% of cases patients were treated by methods not proven effective in real practice (in contrast to artificial conditions of a clinical trial) (22). Importantly, drug efficacy proven in compliance with EBM standards is no warranty that it would help a particular patient. This shifts responsibility for treatment results from the doctor to a professional or regulatory body which issued the corresponding clinical guidelines.

Several reviews point at a major failure of EBM: for example, Miller et al. (23), Every-Palmer et al. (24). Among the proposed causes of failure are conflict of interest introduced by pharmaceutical companies, methodological inconsistency, replacement of clinical thinking by rigid standards, and reductionism. Inadequacy of EBM in real life can be connected with two main factors:

1. Most cases of chronic diseases in real practice require personalized and creative approach from the doctor, as well as patient's active participation in the treatment or lifestyle modification;
2. Despite the fact that drug efficacy in controlling symptoms is formally proven, their ability to manage patient's health in the long term is most often very limited (25).

Remedies used to treat common chronic diseases often fail to help a major part of patients due to obvious pharmacogenetic mechanisms: antidepressants in 38% of cases, asthma medications in 40%, antidiabetic drugs in 43%, arthritis drugs in 50%, drugs for Alzheimer's disease in 70%, anticancer drugs in 75% of cases (26).

On the other hand, in today's economy a pharmaceutical is a commercial product, and its effectiveness is a consumer quality; in this context the drug's inability to meet the needs (to help) a major part of consumers (patients) is unacceptable from the standpoint of market economy.

Thus, in terms of clinical effectiveness of medicinal products, the key tool of medical care, the healthcare systems are definitely underperforming.

With prevalence of diagnosis-centered organizational model, over the last 50 years health systems have shown inability to solve the problem of the most important chronic diseases: cardiovascular, mental, musculoskeletal, cancers, diabetes (27).

### Evidence 5. Opinions of Patients and Health Professionals About Performance of Healthcare System (Russian Experience)

Attitudes of patients and medical professionals in Russia were studied in a WCIOM public opinion poll (2017) (28) and analytical report of Boston Consulting Group (29). As few as 10% of patients gave a positive appraisal of the national healthcare system. A “typical” Russian doctor complains on low professional training, low income with high working load, excess of “paperwork”, professional burnout and low motivation.

Low efficiency of health systems has become a truism both in Russia and high income countries considered as the most technologically advanced. With that, it is necessary to admit that in combating acute and emergency conditions like COVID-19 Russia is performing reasonably well. However, whereas proper organization of acute care and modern technologies reduce mortality in severe cases, they cannot influence the risks related to chronic diseases.

## Question 2. How Does Performance of Healthcare Systems Relate to the Prevailing Scientific Paradigm and Organizational Model of Health Care?

The scientific paradigm and organizational model take center stage in light of the fact that explicit goal-setting and adequate performance KPI are absent in health system in general and medical organizations in particular.

We see the following obvious reasons why the prevailing organizational model limits healthcare performance:

1. As a rule, intervention in the course of a chronic disease takes place at a very late stage: not until a medical diagnosis can be made, and health potential has been depleted for the most part. The objective of mandatory regular medical examinations in occupational medicine is admission to work and detection of occupational and work-related diseases, while prophylactic checkups are designed to reveal early signs of diseases. The data collected during these exams neither enable effective decision making for health promotion, for detecting preclinical signs of health problems nor for solving them. As a result, almost all interventions appear to be reactive, not preventive.

Real individual needs in health improvement extend far beyond medical care, but many aspects are not envisaged by diagnosis-centered model: for example, risk identification, primary prevention through lifestyle change, long-term follow-up at home. On the other hand, high quality complex intervention can eventually be useless if the next step of care lacks continuity.

2. In fighting chronic diseases healthcare system virtually ignores the most effective instruments, namely lifestyle factors: food and nutrition, exercise, stress management, etc. They influence universal mechanisms underlying chronic disease: chronic systemic inflammation, chronic distress, insulin resistance, mitochondrial dysfunction, immune imbalance, etc. (30). Treatment can hardly be effective without these factors being identified and removed.

Many instruments in the diagnosis-centered model are aimed at symptom management, they feature poor benefit/risk ratio; treatment modalities prescribed in line with diagnosis-specific guidelines often ignore personalization.

3. Role distribution in health care specific for the diagnosis-centered model creates predictable deficit of resources: decision making, treatment and burden of responsibility lies on doctors and other health professionals. Patient's initiative, self-organization and responsibility for prevention and treatment results are minimized. At the same time, doctors usually don't perceive their activity as managing health: they are focused on controlling disease symptoms, and most often lack skills in health restoration, improvement and promotion.

Thus, the key limitations of diagnosis-centered organizational model stemming from biomedical paradigm are: reactivity instead of proactivity, use of therapeutic tools only marginally effective in chronic diseases (focus on drugs instead of lifestyle-based prevention), and inevitable scarcity of resources.

4. In Russia there is an additional important factor of health system performing poorly: medical care is only one and probably less significant health determinant. Other, much more important determinants are an interdisciplinary “cocktail” of complex political, economic, social, psychological factors, working and living conditions, education, environment, food supply, traditions, lifestyle, physical activity and exercise, chronic distress, etc.

Already poor performance of health system in Russia dropped further after the corresponding Ministry of health and social affairs had been split in 2012 into Health Ministry and Ministry of Labor and Social Protection, after other health-related functions had been transferred from Health Ministry to external regulatory bodies like Federal Services for surveillance on Consumer rights (Rospotrebnadzor), in Healthcare (Roszdravnadzor), Federal Medical-Biological Agency, and others. This created additional interdepartmental barriers very unfavorable for performance of the whole system.

Distortions in the scientific paradigm and organizational model of healthcare systems has clearly manifested during the COVID-19 pandemic. The high risk groups of severe disease very closely coincide with modifiable risk factors of chronic non-infectious diseases. Hence, COVID-19 long-term prevention and treatment should be built around lifestyle correction and individual behavior (food and nutrition, exercise, sleep, stress management, etc.). However, the public domain very rarely features a system of comprehensive advice for ordinary people. That is, the expert community stays within the diagnosis-centered model of care and focuses on tactic, local preventive tools like vaccination, social distancing, disinfection, personal protective equipment, etc., and neglect true system health improvement.

The diagnosis-centered organizational model does have certain prevention strategies: so-called “risk factor-based prevention”. This is an attempt to target directly certain laboratory or clinical symptoms (high blood cholesterol, high blood pressure, excessive body weight, etc.) to improve disease outcomes. This may work in clinical trials; however, the fact that in large population studies reduction of blood cholesterol with statins or antihypertensive pharmacotherapy can reduce mortality is challenged by some authors. Same is true for mechanistic approach to smoking without considering the psychosocial aspects of this bad habit. (31). Why? Most probably, these “risk factors” are actually risk indicators rather than the cause of higher mortality or morbidity. That is, there is a separate causative factor which influences both mortality and risk indicators. This causative factor is related to psychosocial and lifestyle determinants of health (31). They explain steep growth of mortality from chronic diseases during social and economic crises; they also highlight an urgent need in personalized prevention and addressing the psychosocial factors of health.

## Question 3. How Can Performance of Healthcare System Be Improved Through Shift or Evolution of the Paradigm and Organizational Model?

One of the first public critics of the biomedical paradigm, an American psychiatrist George Engel, proposed an alternative paradigm based on general systems theory, the biopsychosocial model (32, 33). Today this scientific paradigm is actively used in psychology, psychiatry, and rehabilitology (34). The essence of the biopsychosocial paradigm can be expressed in three theses:

1. Human is a complex multilevel hierarchy of nested systems (from molecules, cells, tissues, organs to whole organism, personality, community, and society); within this hierarchy all systems are in structural and functional interconnection;

2. To adequately understand the state of health and the causes of disease one has to integrate information about all levels of human existence: including biological, psychological and social levels;

3. Recovery and maintenance of health requires interventions at all levels of human existence.

The biopsychosocial paradigm suggests a different organizational model of health preservation, person-(patient-)centered, which is free from the problems of diagnosis-centered model. It is designed not to replace but to complement the latter in prevention and treatment of chronic diseases (35).

Here are the principles of person-centered organizational model as we see it:

(1) Interaction with a patient (person) is initiated long before chronic disease. Health preservation and improvement can and should be implemented at any stage of the life cycle, in any point of the wide range of life situations. Participation of health professionals is most natural in the workplace, in educational facility, during medical care (for example, at the stage of rehabilitation), in outpatient setting and during long-term follow up at home.

(2) Health-related interventions are designed to remove negative lifestyle factors contributing to universal mechanisms of chronic diseases under individual circumstances of the particular patient. Instead of managing the symptoms, interventions are focused on the causes and underlying mechanisms, as well as on educating the patient in self-care and self-regulation.

(3) Health professional plays the role of an expert and tutor (coach) helping the person to master health self-management. In difficult cases, a multidisciplinary team of specialists is desirable; decisions on the best interventions are made with direct involvement of the patient, with consideration of individual opportunities and environment.

To scale up the outlined organizational model of health care, there are two prerequisites: IT infrastructure and availability of lifestyle coaches; these are necessary for individual planning and tracking the everyday preventive and therapeutic lifestyle changes.

The person / patient-centered model that actively involves the patient in health recovery and maintenance utilizes inexpensive and cost effective tools for health correction, and thus it saves health system's resources for acute care (27, 36). The worldwide prototype of this model has the format of P4 medicine (37, 38).

A major challenge for wide implementation of P4 medicine approaches is that they rely on doctor's decision making, i.e., inherit the bottleneck of diagnosis-centered model: limited access to a trained professional. Scarcity of doctors certified in P4 medicine makes this option quite expensive worldwide. However, when we focus on early prevention, when patients have as yet only early predictors or mild signs of a chronic condition, a lot could be done through designing a special clinical decision support system (CDSS) for patients. Such a CDSS has to be based on different input data and decision making algorithms than the systems currently in use. Ideally, these data should be available at any time and no cost, and algorithms should rely on robust and valid cause-effect relationship between everyday lifestyle factors and universal mechanisms that underlie age-related diseases. Such relations become increasingly available thanks to development of systems biology.

Prototype data sources for a CDSS for patients are, for example, self-reported tools like suboptimal health status questionnaire SHSQ-25 (39). The authors see it as an “instrument for health measuring in the general population”. Health-related quality of life (QoL) is another relevant metric for well-being at individual and population level. Potentially a questionnaire combining early symptoms of chronic disease and related QoL can be also used to measure the effectiveness of interventions, negative impact of environmental factors, and even performance of health systems.

Another key factor for effective CDSS for patients could be data on individual exposome: lifestyle, nutrition, stress management, toxic exposures, etc. (40). Individual exposome data could be matched with individual signs of suboptimal health and used to propose personal lifestyle modifications (i.e., support decision making at personal level). Exposome data may also be used by public health authorities to plan interventions at population level: run educational campaigns, mitigate social stressors, improve access to fitness facilities, healthy food, etc.

The corresponding questionnaires as data retrieving instruments on health status and exposome still remain to be developed, and without them person-centered healthcare model will hardly be scalable.

The biopsychosocial paradigm and person-centered organizational model should be seen as an essential complement, not an alternative to the prevailing diagnosis-centered model, as a stage of its evolution. In terms of healthcare continuum, the new model is most relevant in health education, in primary prevention, in detection and correction of preclinical health problems, in stress management (to prevent chronic distress), in long-term follow-up after hospital discharge, in rehabilitation, etc. – that is, where the diagnosis-centered model performs poorly.

The new model requires certain important regulatory measures: an increase of public health care expenditures on prevention [today in Russia it amounts to <1% of healthcare budgets, (41)]; economic incentives for all kinds of businesses supportive of individual health recovery and maintenance; regulatory obstacles for businesses that harm public and individual health. Any individual, public and corporate activity aimed at health preservation and promotion should receive regulatory support.

The best mode of introducing the new patient-centered organizational model into practice is changes made simultaneously “top down” (through the regulatory context and infrastructure) and “bottom up” (through local pilot projects, like person-centered health management systems in large corporations, universities, and local communities).

Since diagnosis-centered and person-centered models are very different, the methods of their automation approaches and instruments (required for scaling up) also differ a lot. This is a large separate issue that we have already covered in our review of health management systems (42). Importantly, a promising infrastructural framework for automation of person-centered model can have a form of a digital platform (‘ecosystem’) bringing together three parties: (1) patients (end users), (2) suppliers of products and services necessary for health management, including medical organizations, and (3) providers organizing interaction between end users and suppliers (43, 44).

## Discussion

In this paper we analyze some key problems related to poor performance of health systems with special focus on Russia, EU and USA. We illustrate poor performance by analytical opinions, healthcare costs / lifespan ratio, iatrogenic deaths, effectiveness of pharmaceuticals, and opinions of patients and health professionals.

We found that, surprisingly, the key performance indicators used in healthcare fail to provide adequate input for decision making at every organizational level. Therefore, decision making is based on ‘by default’ conceptual framework: the biomedical paradigm (where the human is equaled to the body) and diagnosis-centered organizational model (where the key in decision making is finding the right diagnosis). Historically they were adequate to address acute conditions and emergencies, but in the face of chronic disease they are inconsistent.

We hypothesize that health system performance cannot be improved without reviewing and revising the scientific paradigm and organizational model that underlie all decision making. In particular, focus on pathophysiology as the essence of disease (biomedical paradigm) should be expanded to involve psychosocial determinants of health and disease (biopsychosocial paradigm). Correspondingly, the current focus on medical diagnosis as the key step in decision making should be expanded to include personalized prevention. Game changers might be the following: addressing person's needs in health management across the whole continuum of care, use of the most effective lifestyle-based preventive interventions, and employment of patient's resources (attention and time) through role reversal. Effective interdisciplinary and interdepartmental efforts at the national level, and allocation of more resources to prevention are also essential, at least in Russia. It also makes sense to think about new key performance indicators for the whole health system.

Is it possible to practice person-centered approach within the framework of biomedical diagnosis-centered model? There are proposals how to improve health system performance through implementation of ‘person-centered care’ model without going beyond the biomedical paradigm (45). However, just declaring person centeredness without actual change of doctors' and patients' worldview, the lifestyle, the role distribution, everyday routines and habits, cannot make a difference in both clinical outcomes and cost effectiveness of care.

The best conceptual and methodological framework for the new organizational model is set by ‘P4 Medicine’. Thanks to new information and communication technologies, the new person-centered model can be scaled, and there are very promising examples of such scaling (46, 47). At the same time, certain essential instruments for person-centered data retrieval remain to be developed: most importantly, on self-reported health status and personal exposome.

## Data Availability Statement

The original contributions presented in the study are included in the article/supplementary material, further inquiries can be directed to the corresponding author/s.

## Author Contributions

AM-P was responsible for the concept, data collection, analysis and interpretation, and drafting the article. DY and MP contributed to the concept, data interpretation, critical revision of the article, and final approval of the version to be published. All authors contributed to the article and approved the submitted version.

## Conflict of Interest

The authors declare that the research was conducted in the absence of any commercial or financial relationships that could be construed as a potential conflict of interest.

## Publisher's Note

All claims expressed in this article are solely those of the authors and do not necessarily represent those of their affiliated organizations, or those of the publisher, the editors and the reviewers. Any product that may be evaluated in this article, or claim that may be made by its manufacturer, is not guaranteed or endorsed by the publisher.
